# State of Affairs of Tuberculosis in Prison Facilities: A Systematic Review of Screening Practices and Recommendations for Best TB Control

**DOI:** 10.1371/journal.pone.0053644

**Published:** 2013-01-25

**Authors:** Natalie V. S. Vinkeles Melchers, Sabine L. van Elsland, Joep M. A. Lange, Martien W. Borgdorff, Jan van den Hombergh

**Affiliations:** 1 Academic Medical Center, Department of Global Health, University of Amsterdam, Amsterdam Institute for Global Health and Development, Amsterdam, The Netherlands; 2 Department of Paediatric Infectious Diseases, Immunology and Rheumatology, VU University Medical Centre, Amsterdam, The Netherlands; 3 Department of Infectious Diseases, Public Health Service (GGD) Amsterdam, Amsterdam, The Netherlands; 4 PharmAccess International, Dar es Salaam, Tanzania; University of Hong Kong, Hong Kong

## Abstract

**Background:**

Prisoners are at high risk of developing tuberculosis (TB), causing morbidity and mortality. Prison facilities encounter many challenges in TB screening procedures and TB control. This review explores screening practices for detection of TB and describes limitations of TB control in prison facilities worldwide.

**Methods:**

A systematic search of online databases (e.g., PubMed and Embase) and conference abstracts was carried out. Research papers describing screening and diagnostic practices among prisoners were included. A total of 52 articles met the inclusion criteria. A meta-analysis of TB prevalence in prison facilities by screening and diagnostic tools was performed.

**Results:**

The most common screening tool was symptom questionnaires (63·5%), mostly reporting presence of cough. Microscopy of sputum with Ziehl-Neelsen staining and solid culture were the most frequently combined diagnostic methods (21·2%). Chest X-ray and tuberculin skin tests were used by 73·1% and 50%, respectively, as either a screening and/or diagnostic tool. Median TB prevalence among prisoners of all included studies was 1,913 cases of TB per 100,000 prisoners (interquartile range [IQR]: 332–3,517). The overall annual median TB incidence was 7·0 cases per 1000 person-years (IQR: 2·7–30·0). Major limitations for successful TB control were inaccuracy of diagnostic algorithms and the lack of adequate laboratory facilities reported by 61·5% of studies. The most frequent recommendation for improving TB control and case detection was to increase screening frequency (73·1%).

**Discussion:**

TB screening algorithms differ by income area and should be adapted to local contexts. In order to control TB, prison facilities must improve laboratory capacity and frequent use of effective screening and diagnostic tools. Sustainable political will and funding are critical to achieve this.

## Introduction

An estimated 8–10 million people are incarcerated worldwide on any given day. Many more are detained for short periods of time [1]. The demographics of the prison population (e.g. low socioeconomic status, large number of migrants, homeless, drug users), in addition to the situational and environmental vulnerabilities of the prison setting (e.g. overcrowding, poor ventilation [2], [3]) increases the risk of contracting tuberculosis (TB) among prisoners. Studies show that TB prevalence rates are up to 83·6 times higher among inmates as compared to the general population [4]. The TB problem affects high (HIC) and middle/low income countries (M/LIC) differently, with an eight times higher TB incidence in M/LIC's prisons [5]. Cost-effective screening algorithms were recently developed and approved by the World Health Organization (WHO) [6], but are more suitable for HIC who can afford light emitting diode (LED) fluorescence microscopy and GeneXpert MTB/RIF® assay [7]. Diagnostic tools vary by prison facility based on the availability of resources and the prevalence of TB, HIV and/or TB/HIV co-infections in the prison setting and community [8]. Screening procedures are therefore adapted to local contexts and may differ greatly between regions. However, Ministries of Health's (MoH) National TB Programmes (NTPs) may still follow international guidelines on TB control in prisons [8], [9]. Screening procedures may be limited, e.g. by prison health staff who are unable to follow standard TB guidelines due to poor training and lack of funding [8]. Other limitations of successful screening practices in prison facilities include the finite available health staff combined with vast numbers of prisoners, hence slow symptom check-ups [8]. Laboratories inside prison facilities are often inadequate or nonexistent, delaying referral of prisoners to outside health services [8]. These limitations lead to high TB rates in prison facilities, likely contributing to transmission to wider communities [10]. Several DNA fingerprinting studies indicated high latent TB infections (LTBI) and active TB among prison contacts [11], [12]. TB in prison facilities is therefore a public health concern not only affecting inmates, but also the wider community [2].

This review aims to explore screening practices and describe TB occurrence by income area and region. As it is yet unclear which screening and/or diagnostic tools are used in prison facilities, this review assists prison services of both HIC and M/LIC to make evidence-based decisions based on actual practice. In addition, it explains challenges to TB control programmes in prisons globally. It will benefit our understanding on tackling these challenges by providing recommendations concerning the most suitable strategies for enhanced TB control in prison facilities.

## Methods

### I. Search strategy

A literature search was conducted for articles published between January 1, 1990 and June 1, 2011 using the online databases PubMed, Embase, Cochrane library, and African Journals Online (AJOL) (see Appendix S1 for detailed search terms). References of selected studies were reviewed to identify additional articles. In addition, abstract databases of selected conference proceedings between January 1, 2010 and June 1, 2011 were searched.

### II. Selection criteria

The PRISMA checklist is attached in Appendix S2. Original research articles or abstracts of studies reporting on screening procedures for detection of TB among prisoners worldwide were included. For inclusion, studies had to have an intervention, cohort or cross-sectional design and full text available in English, French, German or Dutch. Studies published before 1990 were excluded. Articles describing preliminary results or reviews were also excluded, as were studies which did not distinguish between prison populations and other Most-At-Risk-Populations (MARPs) (e.g. migrants, homeless). In addition, studies evaluating treatment monitoring tools were excluded. One reviewer assessed eligibility of all articles meeting the search criteria (NVM), of which randomly one-third were independently assessed by a second reviewer (SvE). Disagreements were resolved by consensus.

### III. Definitions

This review uses several concepts defined as follows:

- Remand prisoners: those who are awaiting trial and short-stay in principle; sentenced prisoners are declared to be guilty of a criminal offense by the verdict of a judge.
- Screening procedure: identification of symptoms and/or signs of unrecognised TB in asymptomatic individuals by the application of a (combination of) method(s) or examination(s) which can be applied rapidly (e.g. symptom questionnaire, chest X-ray (CXR)). A screening test is not intended to be diagnostic and persons with positive findings (termed TB suspects) must be referred for diagnostic procedures that either confirm or refute TB [13].
- Diagnostic procedure: any (combination of) method(s) used to determine LTBI (e.g tuberculin skin test (TST)) or to confirm active TB (e.g. sputum examination) in an individual.
- To make the distinction between low, middle and high income countries, the World Bank [14] definition based on gross national income per capita was used: i) LIC, US$1,005 or less; ii) lower middle income, US$1,006–US$3,975; iii) upper middle income, US$3,976–US$12,275 (MIC); iv) HIC, US$12,276 or more (for the purpose of this study, low and middle income groups were combined into one variable).

### IV. Data extraction

Data extraction was performed on all (NVM) and one-third (SvE) of all included studies by two independent reviewers. Consensus on discordant results was established. Extracted data included information on location(s), year of screening, study design, imprisonment per 100,000 people in the local population, number of participants, basic characteristics of participants, TB prevalence and incidence, LTBI prevalence, TB/human immunodeficiency virus (HIV) prevalence and incidence, HIV prevalence, screening and diagnostic tools applied, frequency of screening, TB suspect criteria, TB case definition, limitations of TB detection and control, and recommendations for improving screening procedures. The results of the methodological quality of studies, using the Downs & Black checklist [15], are presented in Appendix S1.

### V. Data analysis

Analyses were done using Stata version 12·0 (Stata Corporation, TX, USA). Main outcomes were 1) screening and diagnostic procedures (binary); and 2) TB prevalence and incidence (continuous). Main determinants were geographic region (categorical), economic income category (binary), overcrowding in the prison population (binary), routine TB screening (binary), and imprisonment for ≥10 months (binary). A cut-off of 10 months was selected for imprisonment to opt for an approximately equal number of studies in each category. Selection of these main determinants was based on the dispersion in outcome measures by income status and/or geographic regions.

Screening and diagnostic procedures were made binary per tool used. Associations between these outcome measures and the determinants were evaluated using Pearson's chi-squared or Fisher's exact test, as appropriate. Prevalence and incidence data from the studies included in this review was determined by the type of diagnostic tools used within that study. The generalised Kruskal–Wallis test, based on probability-weighted rank-sums, was used to measure the effect of determinants on these skewed outcome data.

A random-effects meta-analysis of TB prevalence in prisons for each summarised screening and diagnostic tool compared to not using that tool was performed, by measuring the total number of TB cases detected by each specific tool. Additionally, a meta-analysis of TB prevalence for each country compared to corresponding general populations was performed. The *I^2^* statistic measures the percentage of residual variability that is attributable to between-study heterogeneity and takes account of the number of studies included in the meta-analysis [16]. The occurrence of heterogeneity across studies was assessed by univariable metaregression to examine potential sources of heterogeneity. Possible effects of overcrowding, screening frequency, screening and diagnostic procedures (i.e. overall, TB symptom questionnaire, CXR, microscopy), implementation of isoniazid preventive therapy (IPT), isolation of TB suspects, and presence/absence of aeration were assessed.

## Results

**Figure 1:**
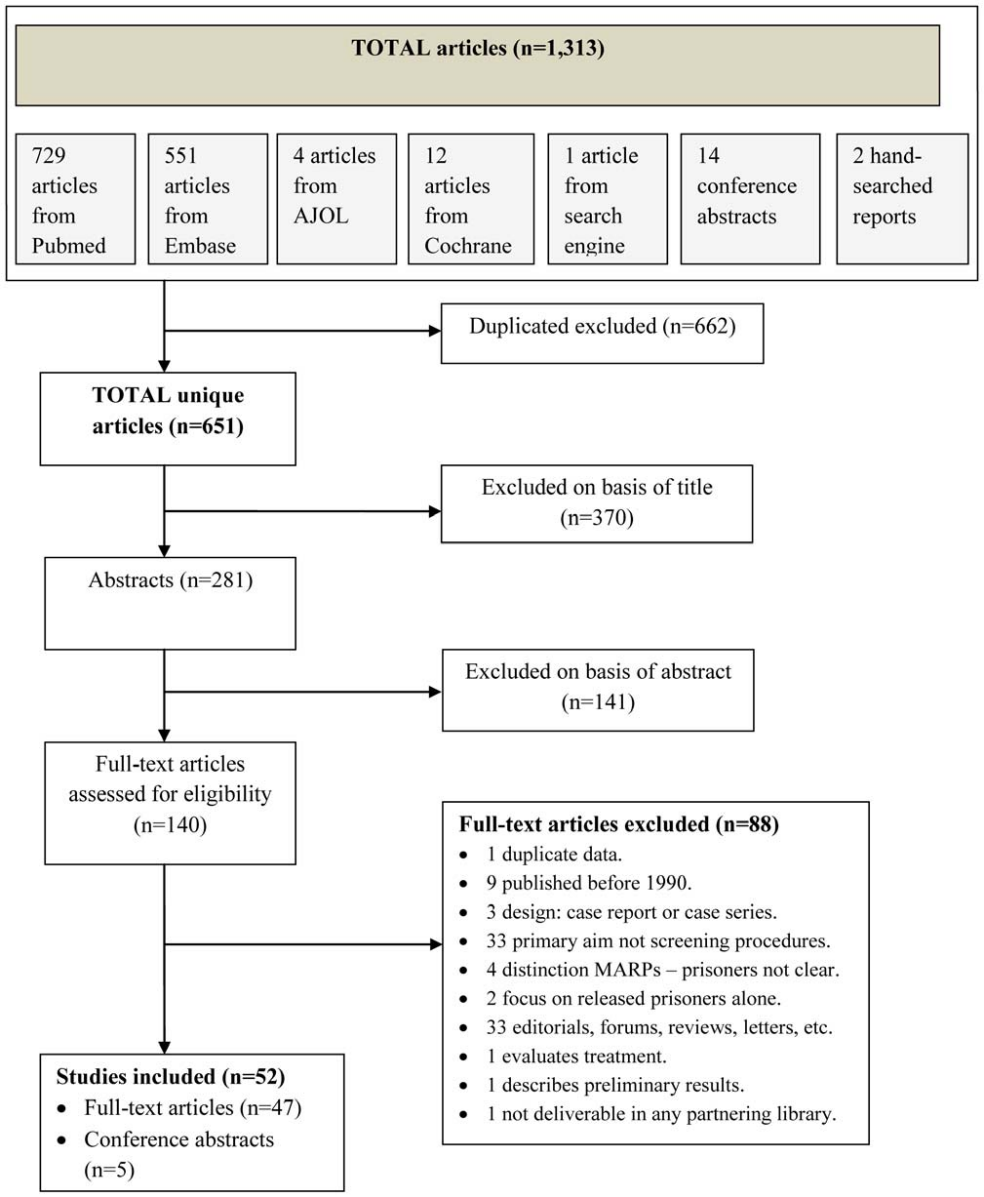
Flow chart of search strategy.

The search strategy identified 651 potential unique articles and abstracts. Based on title and/or abstract, 511 reports were excluded. Of the remaining 140 studies, 52 met the inclusion criteria of which five were conference abstracts (Figure 1). Publication dates ranged from 1993 to 2011 with most publications in 2010 (n = 10). Discordance between reviewers in article selection was less than 20%. Data from a total of 437 prison facilities and 437,430 prisoners who were screened for TB were included in this review.

### I. Study characteristics

A list of all included studies is attached in Appendix S3. Most included studies were from the American region (AMR) (34·6%), mainly the United States of America (USA). The European region (EUR) and African region (AFR) accounted for 17·3% and 15·4% of the included studies respectively. Other studies focussed on the Eastern Mediterranean region (EMR) (13·5%); Western Pacific region (WPR) (11·5%); and the South-East Asian region (SEAR) (7·7%). Selected studies included up to 94 prison facilities, although most studies (57·7%) reported on TB screening in one prison facility alone. Included articles used a cross-sectional (75·0%) or cohort (23·1%) design. Ten studies (19·2%) included both prisoners and prison officers. Seventeen studies (32·7%) reported on both remand and sentenced prisoners, although little data is available on the duration of incarceration for both types of verdicts. The remaining studies did not describe the sentencing status of prisoners.

Eligibility criteria for prisoners to undergo TB screening varied across studies. Twenty-five studies (48·1%) considered for the purpose of their study all prisoners eligible for TB screening, 19·2% screened upon entry into the prison system, and 13·5% screened based on contact with an index case. Six studies (11·5%) excluded prisoners from TB screening if they: i.) were a TB patient at the time of screening, ii.) did not have a cough of ≥2 weeks duration, or iii.) had a history of TB treatment in the past six months.

### II. Characteristics of the prison populations

Average age of prisoners ranged from 29·7 to 31·5 years (n = 37). Most studies included male prisoners only (92·7%), whereas one study focused solely on TB screening among female inmates [17]. Educational level was poor for 74·0% (IQR: 42·9–90·4) of prisoners screened (illiterate or education level less than elementary school). The median of prisoners of foreign ethnicity was 26·7% (IQR: 13·0–41·4) reported by 17 studies. The reported median duration of incarceration was 11·5 months (IQR: 3·2–16·0) (n = 17). Fifteen studies (28·8%) indicated that prison facilities were overcrowded. The reported number of prisoners per cell varied between studies, as did the cell measurements. Overall, studies reported an IQR density range of 33 to 115 prisoners per cell.

### III. Screening procedures

A total of 43 studies reported on the frequency of TB screening in the prison facilities. A large proportion of studies (n = 19/43) reported having screened all prisoners upon admission and thereafter on a regular basis as part of the prison TB programme. Another 16·3% of articles described irregular screening or screening for the purpose of the study only. Seven studies screened all inmates annually, of which one study reported additional active TB screening [18]. One study performed semi-annual TB screening [19]. Passive TB screening was described by 13 studies (30·2%). Twenty-eight studies were conducted in prisons considered to have routine TB programmes, defined as active, annual, semi-annual or entry screening. Studies with routine screening had a median TB prevalence of 343·5 per 100,000 population (IQR: 71–2,714) as compared to a prevalence of 2,227/100,000 (IQR: 705–4,563) for prison facilities without routine TB screening (p = 0·0059). Criteria for identifying TB suspects to be referred for further TB diagnostic work-up were diverse. Most reported criterion (25·0% of studies) was cough for ≥1 week (n = 3/13), ≥2 weeks (n = 4/13), ≥3 weeks (n = 4/13), or any duration (n = 2/13).

All studies described whether screening tools where used to identify TB suspects. About 44% of studies used one screening tool to identify TB suspects, whereas 30·8% of studies reported using two different screening tools. Three studies (5·8%) used three or four screening tools. Medical symptom questionnaires were the most commonly used screening tool to identify TB suspects (63·5%). Among those, 18/33 studies defined a TB suspect based on the presence of cough only (all M/LIC), 12/33 used the presence of at least one TB-symptom, irrespective of cough (i.e. fever, night sweats, anorexia, chest pain and haemoptysis), and 4/33 used a standardised five-symptom questionnaire (cough, sputum production, subjective weight loss, loss of appetite or chest pain). Three studies applied the WHO screening questionnaire, introduced by WHO in 2000 [20]. In addition, the use of chest radiography was frequently reported (36·5%); either conventional CXR (n = 14/19), miniature 70×70 mm CXR (n = 4/19), or digital CXR (n = 1/19). Nine studies described using TSTs in prison screenings, although no consensus was found in the definition of skin indurations' positivity. Positivity ranged from 5 mm response for HIV-seropositive individuals, close contacts of TB patients, BCG-negative individuals or ordinary inmates, to 8 mm, 10 mm or 15 mm reaction in ‘standard’ prisoners. The presence of cough was used predominantly as a screening tool in the AFR (75% of all AFR studies). Use of CXR was uncommon in the AFR (n = 0/8) and EMR (n = 1/6) for TB suspect screening. Table 1 presents the number of studies by screening procedure and income area, and Table 2 describes TB incidence rate by screening and diagnostic instruments.

**Table 1:** Overview of all screening and diagnostic procedures by income area as classified by the World Bank.

		N studies HIC	N studies M/LIC	P-value*	Total number of studies
**Screening procedure**					
	No screening procedure	6 (25·0%)	4 (14·3%)	0·328	10 (19·2%)
	TB symptom questionnaire	2 (8·3%)	2 (7·1%)	0·872	4 (7·7%)
	Presence of at least one TB-symptom	7 (29·2%)	5 (17·9%)	0·335	12 (23·1%)
	Presence of cough (all durations)	1 (4·2%)	16 (57·1%)	<0·0001	17 (32·7%)
	WHO clinical score	0	3 (10·7%)	0·099	3 (5·8%)
	CXR (any type)	10 (40·0%)	9 (32·1%)	0·686	19 (36·5%)
	TST	8 (33·3%)	1 (3·6%)	0·005	9 (17·3%)
**Diagnostic procedure**					
	TST	11 (45·8%)	6 (21·4%)	0·061	17 (32·7%)
	Microscopy (ZN and LED)	14 (58·3%)	26 (89·7%)	0·010	40 (76·9%)
	Solid culture	14 (58·3%)	20 (71·4%)	0·322	34 (65·4%)
	CXR (all types)	13 (54·2%)	6 (21·4%)	0·015	19 (36·5%)
	Drug susceptibility testing	1 (4·2%)	10 (35·7%)	0·005	11 (21·2%)
**Total**					**52 (100%)**

* P-value derived from the Wald Test for the association between a screening procedure and income area.

Source: [14].

**Table 2:** Incidence rates of active TB (%) and prison screening and diagnostic procedures by income area as classified by the World Bank.

Income category	Author, year (country)	Screening period	Screening procedures	Diagnostic procedures	Cases, n (at risk)	Incidence rate
**Middle/Low income countries**	Ferreira et al., 1996 (Brazil)	1992–1993	Presence of at least one TB symptom	TST, CXR, ZN microscopy, LJ culture	4 (559)	0.72%
	Habeenzu et al., 2007 (Zambia)	2000–2001	None	LED microscopy, LJ culture	245 (6,118)	4.01%
	Sanchez et al., 2010 (Brazil)(a)	NA	CXR, Presence of cough only (≧3 weeks)	ZN microscopy, LJ culture	NA	4.82%
	Sanchez et al., 2010 (Brazil)	NA	CXR, Presence of cough only (≧3 weeks)	ZN microscopy, LJ culture	NA	7.90%
	Sanchez et al., 2009 (Brazil)	2005–2005	CXR	ZN microscopy, LJ culture	34 (1,696)	2.00%
	Sanchez et al., 2005 (Brazil)	2002–2002	CXR, Presence of at least one TB symptom, WHO score	TST, CXR, ZN microscopy, LJ culture, DST	32 (1,052)	3.04%
**High income countries**	Chiang et al., 2002 (Taiwan)	1998–1999	Mobile miniature CXR	ZN microscopy, LJ culture, CXR	88 (51,496)	0.17%
	Jones et al., 1999 (USA)	1995–1997	CXR	TST, ZN microscopy	38 (∼13,239*)	0.27%
	Koo et al., 1997 (USA)	1989–1991	TST	ZN microscopy, LJ culture, CXR	10 (5,421)	0.18%
	Leung et al., 2005 (Hong Kong SAR, China)	2001–2003	Presence of at least one TB symptom, CXR	ZN microscopy, LJ culture	10 (814)	1.23%
	Martin Sanchez et al., 2001 (Spain)	1991–1999	CXR, TST	ZN microscopy, LJ culture	6 (2,541)	0.24%
	Mor et al., 2008 (Israel)	1998–2004	Presence of at least one TB symptom, TST	ZN microscopy, LJ culture, CXR, DST	23 (NA)	0.03%
	Saunders et al., 2001 (USA)	1998–1999	Presence of at least one TB symptom, TST	ZN microscopy, LJ culture, CXR, bronchoscopy or thoracotomy	60 (∼14,109*)	0.42%

*Note:* NA: Not available;

* Data not precisely available.

Source: [14].

### IV. Diagnostic procedures

Forty-five studies (86·5%) described TB case definitions. Most common definitions were: ‘any individual with at least one smear microscopy examination positive for acid-fast bacilli or a positive culture’ (42·3%), and ‘any individual with radiological signs such as the presence of infiltrates, cavitations, solitary nodules, fibrotic lesions and pleural effusions suggestive of active TB disease’ (21·2%). Twenty-five studies (48·1%) used a combination of different case definitions for TB diagnosis.

Ten studies (19·2%) described the use of one tool to diagnose latent or active TB, whereas the majority (55·8%) diagnosed inmates based on two different tools. Three diagnostic tools were used in 15·4% of studies and 9·6% diagnosed TB using four tools. These tools included TST, smear microscopy, solid culture, and CXR. Bacteriological confirmation by microscopic Ziehl-Neelsen (ZN) staining was used in the majority of studies (75·0%). ZN staining was frequently combined with other diagnostic tools; 21·2% in combination with Löwenstein-Jensen (LJ) culture; 13·5% in combination with LJ culture and drug susceptibility testing (DST). Another 25·0% of studies combined microscopy with chest radiography, among other tools. Two studies included broncho-alveolar lavage (BAL). None of the included studies applied rapid serology tests for TB based on antibody detection. Type and combination of tools used to diagnose TB varied by region and income area. TST was frequently used by WPR (n = 3/6) and AMR (n = 8/18) for diagnostic work-up. The SEAR did not use CXR at all, only one AFR study used the tool (n = 1/8), and AMR used chest radiography in 50% of studies. The EMR applied little solid culture (n = 2/7); and, all studies from SEAR and AFR used ZN or LED microscopy for TB diagnosis.

### V. Measures of tuberculosis occurrence

**Figure 2:**
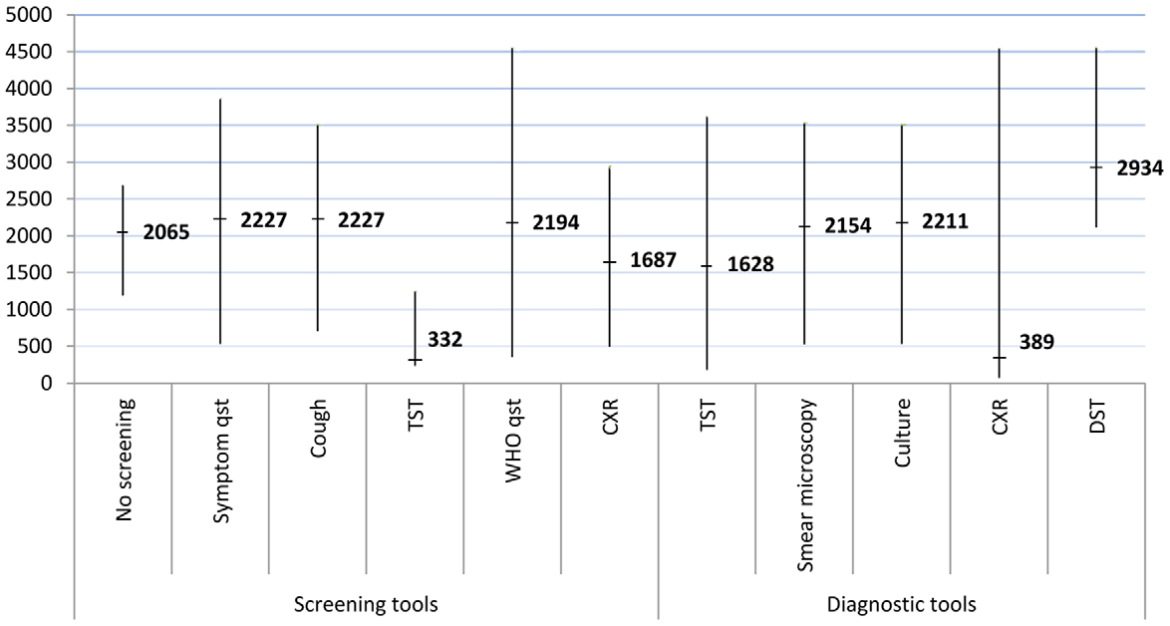
Plot illustrating the median and interquartile range of TB prevalence per 100,000 prison population for different screening and diagnostic tools used in prison facilities.

Forty-seven studies (90·4%) reported on prevalence of active TB with an overall median prevalence of 1,913 cases of TB per 100,000 prisoners (IQR: 332–3,517). Table 3 shows prevalence and incidence of TB and TB/HIV co-infection. By region, the AFR had the highest median prevalence of 3,357 cases of TB per 100,000 prison population (IQR: 1,551–4,354) (p = 0·15), which was concentrated in southern Africa. A significantly lower median prevalence is described for North America (all USA) (180/100,000; IQR: 60–332) as compared to other regions globally (p = 0·0002). Similarly, the western EUR reported a lower median prevalence of TB as compared to other regions (490/100,000; IQR: 243–1,687) (p = 0·07). M/LIC showed a substantially higher median prevalence of active TB (2,712/100,000; IQR: 1,763–4,563) as compared to HIC (289/100,000; IQR: 71–897) (p = 0·0001). Prevalence of active disease was higher among prisoners incarcerated for a median of 10 months or longer (4,354/100,000; IQR: 2,580–6,818) as compared to less than 10 months (2,053/100,000; IQR: 1,763–3,197) (p = 0·039). The overall odds ratio (OR) for prevalence of active TB by income was 33·58 (95%CI: 16·35, 68·97). There was substantial between-study heterogeneity. The overall *I* ^2^ statistic was 99·7% (95%CI: 96·4, 100). The heterogeneity did not differ after stratification by country income: the *I* ^2^ for both HIC and M/LIC was 99·7%. No other variables (e.g. overcrowding, IPT) decreased the between-study variance in univariable metaregression. The odds of TB prevalence was highest in prison facilities not using a screening tool for detecting TB suspects (OR: 15·9; 95%CI: 14·0, 18·1). TB symptom questionnaires (OR: 3·7; 95%CI: 3·4, 4·0) and smear microscopy (OR: 7·1; 95CI%: 6·2, 8·2) were also found to have a higher odds of TB prevalence. There was extensive heterogeneity between the different tools with an overall *I^2^* statistic of 99·9% (95%CI: 94·6, 100). Both cough as a screening tool and TB symptom questionnaires were found to have a high median prevalence of 2,227 cases per 100,000 prison population (IQR: 705–3,517 and IQR: 534–3,886, respectively) (Figure 2). This data corresponds to the results of the meta-analysis. In addition to smear microscopy, solid culture (2,211/100,000 population; IQR: 534–3,517) and DST (2,934/100,000 population; IQR: 2,114–4,563) had highest reported median prevalence of TB.

**Table 3:** Measures of TB occurrence by WHO region and income area according to the World Bank classification (median; interquartile range).

TB occurrence	High income countries				Middle/Low income countries						Overall
	AMR	EUR	EMR	WPR	AMR	AFR	SEAR	EUR	EMR	WPR	
**TB prevalence (per 100,000)**	179 (59–332)	446 (236–1,260)	6,250*	3,339 (258–6,421)	2,935 (2,065–5,714)	3,357 (1,551–4,354)	1,397 (461–2,580)	4,054 (2,114–5,995)	2,194 (657–3,886)	2,551*	1,913 (332–3,517)
**TB incidence (per 1000PY)**	2·7 (1·8–4·2)	6·4*	0·3*	7·0 (1·7–12·3)	30·0 (20·0–48·2)	40·1*	.	.	.	.	7·0 (2·7–30·0)
**Latent TB infection (%)**	12·8 (1·6–26·9)	36·5 (10·1–55·9)	24·2*	9·5 (1·2–22·1)	56·6 (53·6–59·7)	37·5*	.	.	24·1 (3·1–45·2)	.	17·9 (3·0–33·6)
**TB/HIV prevalence (%)**	16·6 (12·0–24·5)	7·0 (5·1–13·2)	0*	.	9·6 (4·6–14·6)	25·0 (15·1–36·0)	37·5*	.	3·2*	.	13·2 (5·1–25·0)
**HIV prevalence (%)**	5·7 (1·6–9·0)	9·0 (7·0–15·1)	.	0·2 (0–0·3)	24·6*	24·4 (11·3–30·0)	39·5*	.	2·0*	.	9·0 (2·0–24·4)

*Note*:

* data available from one study.

Source: [14].

Thirteen studies (25·0%) reported the incidence of active TB, with a median of 7·0 cases of TB per 1000 person-years (PY). None of the articles from the SEAR reported incidence of active TB. Among the 13 studies, incidence of TB ranged from 0·25 to 40·0 per 1000PY reported in EMR [21] and AFR [22] studies, respectively. Reports from South America showed a considerably higher TB incidence (30/1000PY; IQR: 20–48) as compared to other regions worldwide (p = 0·019). Although HIC reported a lower median incidence of active TB (5·3/1000PY; IQR: 1·8–20·0) as compared to M/LIC (12·3/1000PY; IQR: 2·7–40·1), the difference has likely arisen by chance (p = 0·50). Studies including cough as a screening tool reported nine times higher incidence of TB compared to studies which did not, which was not attributable to a difference in income area (all studies from M/LIC). Similarly, studies using CXR as a screening method reported a 3.8 times higher incidence of TB compared to studies using other screening tools. TB incidence was 2·1 times higher in prison facilities without entry screening than in those with entry screening.

Table 3 summarises the overall median prevalence of LTBI among prisoners from 24 studies (median: 17·9%; IQR: 3·0–33·6). Data from South America (two studies) reported the highest median prevalence of 56·6% (IQR: 53·6–59·7) as compared to other regions worldwide (p = 0·037) [17], [23]. Highest LTBI prevalence was reported in a study on female inmates (median: 59·7%) [17]. The WPR reported the lowest median prevalence of 9·5% (IQR: 1·2–22·1). There was some evidence that LTBI prevalence was higher in M/LIC (45·2%; IQR: 37·5–53·6) than in HIC (17·4%; IQR: 2·8–26·9) (p = 0·043).

Prevalence of TB/HIV co-infection was reported by 17/52 studies. The overall median TB/HIV prevalence in prison facilities globally was 13·2% (IQR: 5·1–25·0) (see Table 3), ranging from 0·0% to 37·5% in the EMR [21] and SEAR [18], respectively. Nonetheless, it may be questioned to what extent inmates were tested for HIV. There was little difference in TB/HIV prevalence between M/LIC (15·1%; IQR: 4·6–36·0) and HIC (12·0%; IQR: 6·0–16·6) (p = 0·34). Twenty-one studies performed serology for HIV and found an overall prevalence of 9·0% (IQR: 2·0–24·4), ranging from 0·2% to 39·5% in the WPR [24], [25] and SEAR [26], respectively. A large proportion of studies came from the USA (n = 6/21). More studies from HIC tested prisoners for HIV (n = 13) than M/LIC (n = 8), with M/LIC reporting a 3·5 times higher HIV prevalence (24·5%; IQR: 9·5–34·8) compared to HIC (7·0%; IQR: 1·0–9·0) (p = 0·017).

### VI. TB control limitations

The included studies reported a wide range of limitations for TB control, as provided in Table 4. Most studies (61·5%) described limited accuracy of diagnostic algorithms and lack of adequate laboratory facilities (e.g. no HIV testing, no radiography equipment) as limitations. Logistic and financial constraints were reported by 57·7% of studies, including frequent movement of prisoners between the prison system and community, budget constraints of the prison facility, poor access to health care, and prohibition to attend local clinics or hospitals for ‘security’ reasons.

**Table 4:** Limitations of current TB control programmes in prison facilities.

• Limited accuracy of diagnostic algorithms and lack of adequate laboratory facilities, as well as frail TB screening tools.
• Inadequate financing and logistic accomplishments, consequently from lack of political priority of prison environments and prisoner health.
• Lack of well-organised health services, including poorly coordinated and supervised prison health services and lack of motivated prison medical staff.
• Poorly controlled treatment services and supervision; delays in diagnosis resulting in deferment of treatment for both TB and HIV infection.
• High-risk prison environment with little attention to institutional vulnerabilities (e.g. overcrowding, ventilation) and fragile populations (e.g. female inmates, foreign-born inmates).

Other reported limitations include i) the lack of well-organised health services (including lack of skilled and motivated manpower or adequate referral services) (21·2%); ii) poorly controlled treatment services and supervision (including non-compliance) (23·1%); and iii) high-risk environmental factors (overcrowding and/or poor ventilation and language and cultural barriers for foreign-born inmates) (19·2%).

### VII. Recommendations for improving TB control programmes

Recommendations for improving TB control in prison facilities were provided by 90·4% of the articles, most of which recommended regular screening of prisoners for active and latent TB (73·1%). Suggestions for achieving this goal included: i) early detection by active, regular and systematic search for TB cases among prisoners; ii) screening upon entry; and iii) TB screening for all (prospective) prison employees.

In addition, the need for improved TB and TB/HIV case management was stressed (69·2%). Recommendations to achieve this included: i) the provision of treatment and improved treatment adherence; ii) follow-up of those transferred between prison facilities or after release into the community; and iii) IPT among high-risk groups, including HIV-infected prisoners, clinical staff and guards.

Additional recommendations to enhance TB control in prison settings included: i) increased and improved interventions to prevent (further) transmission (55·8%) (such as improved ventilation); ii) the necessity of validated screening algorithms and tools (53·9%) (including administering CXR screening tests to all inmates and/or TB suspects and implementing a medical symptom questionnaire); iii) logistic and policy improvement (51·9%) (establishing TB diagnostic and management units for coordination and implementation of efficient information systems that involve all prison facilities and NGOs in the country, and increasing political commitment); and iv) advancing efforts to identify high-risk groups tailored to the local epidemiology of TB, with a specific focus on immigrants, refugees and female inmates (23·1%).

## Discussion

Fifty-two articles were identified describing screening and diagnostic procedures in prison facilities. This review found high prevalence and incidence of TB, HIV and TB/HIV co-infections, providing evidence that these infections are substantially elevated in prison facilities compared to general populations [27], urging awareness among policy makers to prioritise prisoners. Although TB prevalence and incidence was higher in facilities without entry screening as well as among inmates incarcerated for 10 months or longer, it is unclear to what extent these figures are attributable to confinement. It was beyond the scope of this review to address specific risk factors of TB prevalence and incidence. This review may be biased by several factors, including biases associated with publication, study design (cohort and cross-sectional), study implementation, or measurement error.

Medical symptom questionnaires (63·5%) and chest radiography (34·6%) were most commonly used for TB suspect identification in prison facilities. Symptom questionnaires were the main screening tool to identify TB suspects in M/LIC (75·0% of all M/LIC studies). WHO recommends symptom questionnaires to be adapted to local conditions [20]. This review suggests that the application of CXRs and symptom questionnaires, including the presence of cough, yields better detection of (new) TB cases, probably by finding radiographic signs or symptoms in otherwise unrecognised cases. For M/LIC, use of CXRs in addition to symptom questionnaires will increase sensitivity of screening algorithms [28]. Local governments of M/LIC will need to invest in upgrading prison health system infrastructure to incorporate CXRs.

Microscopic ZN staining and LJ culture were the most frequently applied diagnostic tools, and more often applied in M/LIC compared to HIC. The distribution of TB prevalence by screening procedure suggests that smear microscopy, solid culture, and DST have the highest impact on diagnosing TB cases in prison facilities. However, it is possible that prison facilities experiencing high TB rates acquired more often smear microscopes and/or culture equipment compared to prison facilities with less TB occurrence. The meta-analysis experienced substantial heterogeneity (99·9%; 95%CI: 94·6, 100); these results should be interpreted with caution as high dispersion is a source of error. All studies originating from SEAR and AFR used smear microscopy, yet not all studies from these regions confirmed smear-negative cases with culture. A recent study at a Zambian prison found high rates of culture-confirmed cases in asymptomatic or smear-negative individuals, or in those with normal CXRs [29]. Therefore, WHO recommends sputum examination for all TB suspects to confirm TB diagnosis [20]. Microbacteriological screening (i.e. sputum examination, culture) should be performed regardless of the absence of symptoms [30], [31], [32], especially at prison facilities with high TB/HIV co-infection, where patients are asymptomatic or have minor symptoms. Consequently, both diagnostic tools are a cornerstone in prison screening algorithms for HIC and M/LIC, although upgrading techniques to LED microscopy and liquid culture, in addition to DST, is indispensable [30], [33]. Conventional or digital CXRs are used as a diagnostic tool in 36·5% of included studies. However, these methods are costly, have limited availability in most settings, and results are difficult to interpret [8], [34]. This may explain why this review found increased use of CXRs in HIC (EUR, WPR and AMR). TSTs are suitable tests to assess the prevalence of LTBI and may predict the risk of active TB in prison populations and staff [35], hence identifying high-risk individuals eligible for IPT (e.g. HIV-infected inmates, contacts of TB index cases). TSTs are mainly applied by HIC, both for screening and diagnostic procedures. This review found that 50% of studies from WPR applied TSTs in diagnostic procedures. The WPR concurrently had the lowest median LTBI prevalence of 9·5% (IQR: 1·2%–22·1%), potentially suggesting that regular application of TSTs could increase detection of LTBI, thereby stabilising overall LTBI over time [36]. The use of TSTs in diagnosing active TB is however discouraged by WHO [35]. New point-of-care TB tests could benefit prison facilities globally, especially in settings with limited electricity and extreme temperatures. The feasibility of the GeneXpert MTB/RIF for routine point-of-care diagnosis of TB in prison clinics of resource-limited settings is still questionable [37], as its scaling-up and maintenance bears high costs and requires system strengthening [38], [39].

Limited accuracy of diagnostic algorithms and lack of adequate laboratory facilities (61·5%) were identified as key limitations for TB control programmes in prisons. Some prison settings allowed diagnostic testing and medical follow-up only for those presenting with symptoms [18], [22], [40], [41], while quality control of smear examinations is not always present in local laboratories [42]. Lack of well-organised health services or adequate referral was mentioned by 21·2% studies. Poor health services may be related to limited infrastructure, equipment, staff or transport, as well as incomplete medical information systems and out-of-pocket payments [19], [22], [40], [43], [44]. Weak prison infrastructure limits infection control and adequate isolation of cases [45], but also neglects hard-to-reach populations (e.g. female and/or foreign-borne inmates) who may encounter violence, discrimination or language barriers [24], [46], [47], [48], [49].

Table 5 describes critical steps for enhancing TB control in prisons, based on recommendations provided by the included studies. Recommendations focused on improving TB case detection for latent and active TB in prison facilities through more frequent screening and passive and active case-finding of inmates (73·1%). This review found a higher TB prevalence in prison facilities without routine TB programmes (p = 0·0059), suggesting that screening at regular intervals potentially decreases overall TB prevalence, although this association may be due to other factors. Regardless, increased TB screening will lead to increased diagnosis and, if treated, then reduced TB prevalence. Entry and regular screening, as well as self-referral for all inmates, medical staff and prison guards should therefore be promoted [50], [51], [52], [53]. Improving infrastructure and enhancing diagnostic procedures are crucial for TB control in prison facilities and should be combined with strong political and financial commitments, high-level involvement and support of national and international stakeholders [54]. The need for increased sustainable and effective investment in health systems and services from both private and public sectors (Millennium Development Goal 8) is herewith highlighted [55]. Improvements of prison health systems and incarceration conditions need to be prioritised when aiming at successfully controlling TB in these high-risk settings.

**Table 5:** Steps to enhance TB control in prison facilities for both high and middle/low income countries.

More regular and rapid screening of prisoners for active TB and LTBI for early diagnosis
1) Entry screening for all inmates, at least TB symptoms screening questionnaires upon admission.
2) TB screening for all (prospective) prison employees.
3) Performance of active, regular and systematic search for new TB patients during incarceration.
4) Self-referral for inmates, clinical staff and prison guards with TB symptoms.

Need for improved TB and TB/HIV case management
1) The provision of adequate treatment for TB, HIV and TB/HIV co-infections.
2) Increasing the effectiveness of treatment (directly observed) as well as adherence of patients.
3) Medical follow-up of those transferred between prison facilities or after release into the community.
4) TST implementation and IPT provision for high-risk groups, including HIV-infected prisoners, staff and guards. Efficiently rule out active TB and monitor side-effects.
5) Continuous training programmes for both staff and prisoners (educational programmes).
6) Provider-initiated (free) provision of voluntary counseling and testing for the detection of HIV infection.

Increased and improved interventions to prevent (further) transmission
1) Improvements of confinement and environmental vulnerabilities such as ventilation conditions.
2) Decrease overcrowded prison cell environments for male, female, refugee, and foreign-borne inmates.
3) Provision of isolation facilities for patients and protective gear for medical staff.
4) Ensure TB screening on exit to prevent disease transmission from prison to community.

The necessity of validated screening algorithms and tools
1) Administering CXR screening tests to all inmates and/or TB suspects.
2) Introducing and implementing a medical symptom questionnaire for all inmates.
3) Use of DST and strain identification for detecting drug resistance.
4) Development of a rapid and accurate point-of-care TB test suitable for low-resource prison settings.
5) Introduction of sputum smear microscopy services in all prison clinics.
6) Use of standard sputum culture to detect smear-negative TB cases.

Logistic and policy improvement
1) Increase national political commitment, including funding.
2) Good governance, including good strategies to find and treat TB cases.
3) Adherence of established (international) infection control policies.
4) Stimulate greater monetary and technical support from international donors to increase TB prison screening leverage, sustainability and consistency with international standards.
5) Establishing a prison TB diagnostic and management unit for coordination; implementation of an efficient information system involving all prison facilities and NGO's.

## Supporting Information

Appendix S1 **Detailed methods.**
(DOCX)

Appendix S2 **PRISMA 2009 Checklist.**
(DOCX)

Appendix S3 **Studies included in this review, reporting on TB screening procedures in prison facilities.**
(DOCX)
