# Supplementary material for: State of Affairs of Tuberculosis in Prison Facilities: A Systematic Review of Screening Practices and Recommendations for Best TB Control
Source: PLoS One. 2013 Jan 25;8(1):e53644. doi: 10.1371/journal.pone.0053644 (PMC3556085; doi:10.1371/journal.pone.0053644)
Supplement: Appendix S1 — Detailed methods. (DOCX) [file pone.0053644.s001.docx]

**Appendix 1**

**Detailed methods**

**Search strategy**

# A literature search was conducted for articles available between January 1, 1990 and June 1, 2011 using the online databases PubMed, Embase, Cochrane library, and AJOL. The search terms were: “tuberculosis” [Mesh] or “tuberculosis” AND “prisons” or “prison*” or “jail*” or “inmate*” or “correctional” or “penitentiar*” or “imprison*” or “penal institution*” NOT case reports OR review. References of selected articles were reviewed to identify additional eligible articles. In addition, abstract databases of selected conference proceedings between January 1, 2010 and June 1, 2011 were searched: the 41st Union World Conference on Lung Health 2010, the 18^th^ Conference on Retroviruses and Opportunistic Infections in 2011, the World Association of Sarcoidosis and Other Granulomatous Disorders 2011, among others. No review protocol was published.

**Assessment of methodological validity**

Two reviewers independently evaluated the quality of all (NVM) and a third (SvE) of all included studies using the Downs & Black checklist [1]. An adapted version was used to grade reporting quality, internal and external validity. The outcome measure derived from this checklist ranges between 1 to 13 points. The reporting quality was measured by a score between 0-6 points, where a score of 0-1 was considered low, 2-4 average, and 5-6 considered high quality. External validity was measured by a score of 0, 1 or 2 points, considered low, average, and high quality respectively. Internal validity was scored between 0-5 points, where 0-1 was considered low, 2-3 average and 4-5 a high score. Five abstracts were excluded from the quality assessment process.

**Study validity**

The quality of reporting was mediocre; 50·0% of all studies were average quality, 35·4% were high quality. External validity was average and high for the majority of the articles (87·5%). Forty-five (86·5%) articles scored average and high on internal validity. Initial between-reviewer variability was 20%, disagreements were solved by consensus.

**References**

1. Downs SH, Black N (1998) The feasibility of creating a checklist for the assessment of the methodological quality both of randomised and non-randomised studies of health care interventions. J Epidemiol Community Health 52: 377-384.
