# Supplementary material for: State of Affairs of Tuberculosis in Prison Facilities: A Systematic Review of Screening Practices and Recommendations for Best TB Control
Source: PLoS One. 2013 Jan 25;8(1):e53644. doi: 10.1371/journal.pone.0053644 (PMC3556085; doi:10.1371/journal.pone.0053644)
Supplement: Appendix S3 — Studies included in this review, reporting on TB screening procedures in prison facilities. (DOCX) [file pone.0053644.s003.docx]

**Appendix 3**

**Studies included in this review, reporting on TB screening procedures in prison facilities.**

| **Author, Year (country)** | **Screening Period** | **Cases screened, *n* (At Risk)** | **Prevalence of active TB in prisons x 100,000** | **Prevalence in general population x 100,000 ^(a)^** | **Prevalence Rate Difference** | **Prevalence of LTBI in prisons per 100,000** |
| --- | --- | --- | --- | --- | --- | --- |
| **Abebe et al., 2011 (Ethiopia) [1]** | 2008 - 2008 | 371 | 1,913 | 432 | 1,481 | NA |
| **Abrahao et al., 2006 (Brazil) [2]** | 2000 - 2001 | 1,052 | 2,065 | 78 | 1,987 | 53,565 |
| **Adib et al., 1999 (Lebanon, Rep.) [3]** | 1995 – 1995 | 3,931 | 7,386 | 40 | 7,346 | 45,179 |
| **Aerts et al., 2000 (Georgia) [4]** | 1997 - 1998 | 7,473 | 5,995 | 136 | 5,859 | NA |
| **Assefzadeh et al., 2009 (Iran, Rep) [5]** | 2004 - 2005 | 768 | 583 | 42 | 541 | NA |
| **Banda et al., 2009 (Malawi) [6]** | 2005 - 2005 | 7,661 | 705 | 265 | 440 | NA |
| **Banu et al., 2010 (Bangladesh) [7]** | 2005 - 2007 | 11,000 | 2,227 | 440 | 1,787 | NA |
| **Borja et al., 2010 (Philippines) [8]** | NR | 2,666 | 2,551 | 520 | 2,031 | NA |
| **Butler et al., 1999 (Australia) [9]** | 1996 - 1996 | 789 | NA | 7.9 | NA | 1,200 |
| **Carbonara et al., 2005 (Italy) [10]** | 2001 - 2002 | 1,247 | 446 | 11 | 435 | 17,411 |
| **CDC 2003 (Botswana) [11]** | 2002 - 2002 | 1,290 | 3,566 | 637 | 2,929 | NA |
| **Chiang et al., 2002 (Taiwan) [12]** | 1998 - 1999 | 51,494 | 259 | 181 | 77.7 | NA |
| **Chigbu et al., 2010 (Nigeria) [13]** | 2006 - 2006 | 168 | 1,190 | 265 | 925 | 37,500 |
| **Ferreira et al., 1996 (Brazil) ^#^[14]** | 1992 - 1993 | 350 | 5,714 | 103 | 5,611 | 59,714 |
| **GGD Nederland 2010 (Netherlands) [15]** | 2009 - 2009 | 38,061 | 70 | 8.9 | 61.1 | NR |
| **Habeenzu et al., 2007 (Zambia) [16]** | 2000-2001 | 1,080 | 22,685 | 520 | 22,165 | NA |
| **Hussain et al., 2003 (Pakistan) [17]** | 2001 - 2001 | 425 | NA | 551 | NA | 3,088 |
| **Jittimanee et al., 2007 (Thailand) [18]** | 2004 - 2005 | 71,594 | 355 | 191 | 164 | NA |
| **Johnsen 1993 (USA) [19]** | 1990 - 1990 | 1,306 | 493 | 14 | 479 | 629 |
| **Jones et al., 1999 (USA) [20]** | 1995 - 1997 | 10,110 | NR | 12 | NA | 1,593 |
| **Karabela et al., 2010 (Greece) [21]** | 2005 - 2009 | 190 | 250 | 8.2 | 241.8 | NA |
| **Kazi et al., 2010 (Pakistan) [22]** | 2007 - 2008 | 384 | 2,194 | 394 | 1,800 | NA |
| **Kiter et al., 2003 (Turkey) [23]** | 1997 - 2001 | 615 | 2,114 | 55 | 2,059 | NA |
| **Koo et al., 1997 (USA) [24]** | 1991 - 1991 | 2,944 | 332 | 14 | 318 | 29,654 |
| **Leung et al., 2005 (Hong Kong SAR, China) [25]** | 2001 - 2001 | 814 | 6,421 | 155 | 6,266 | NA |
| **Levy et al., 2007 (Australia) [26]** | 1996 & 2001 | 914 | NA | 7.6 | NA | 22,115 |
| **MacIntyre et al., 1999 (Australia) [27]** | 1997 - 1997 | 190 | NA | 8.4 | NA | 9,474 |
| **MacIntyre et al., 1997 (USA) [28]** | 1994 - 1994 | 2,606 | NA | 13 | NA | 18,108 |
| **Martin Sanchez et al., 1994 (Spain) [29]** | 1989 - 1990 | 729 | 2,707 | 25 | 2,682 | 56,189 |
| **Martin Sanchez et al., 1995 (Spain) [30]** | 1991 - 1992 | 1,232 | 1,260 | 32 | 1,228 | 55,535 |
| **Martin Sanchez et al., 2001 (Spain) [31]** | 1991 - 1999 | 2,541 | 236 | 32 | 204 | 2,848 |
| **McLaughlin et al., 2003 (USA) [32]** | 1999 - 1999 | 294 | 2,721 | 8.6 | 2,712 | 27,891 |
| **Mor et al., 2008 (Israel) [33]** | 1998 - 2004 | 368 | 6,250 | 11 | 6,239 | 24,179 |
| **Nduaguba et al., 2010 (USA) [34]** | 2006 - 2007 | 1,274 | 0 | 6.3 | -6.3 | 785 |
| **Noeske et al., 2006 (Cameroon) [35]** | 2003 - 2004 | 2,474 | 3,517 | 250 | 3,267 | NA |
| **Noeske et al., 2011 (Cameroon) [36]** | 2009 - 2009 | 3,219 | 3,197 | 190 | 3,007 | NA |
| **Nyangulu et al., 1997 (Malawi) [37]** | 1996 - 1996 | 914 | 5,142 | 351 | 4,791 | NA |
| **Payanandana et al., 2002 (Thailand) [38]** | 1998 - 2000 | 5,283 | 2,934 | 228 | 2,706 | NA |
| **Puisis et al., 1996 (USA) [39]** | 1992 - 1994 | 126,608 | 68 | 14 | 54 | 17,689 |
| **Rao 2004 (Pakistan) [40]** | 2002 - 2002 | 4,870 | 657 | 535 | 122 | NA |
| **Rutz et al., 2008 (USA) [41]** | 2002 - 2003 | 11,576 | 9 | 7 | 2 | 2,972 |
| **Sanchez et al., 2010 (Brazil) [42]** | NR | 785 | NR | 50 | NA | NA |
| **Sanchez et al., 2005 (Brazil) [43]** | 2002 - 2002 | 1,081 | 4,563 | 71 | 4,492 | NR |
| **Sanchez et al., 2009 (Brazil) [44]** | 2005 - 2005 | 1,696 | 2,712 | 57 | 2,655 | NA |
| **Sanchez et al., 2010 (Brazil) [45]** | NR | 4,326 | 2,935 | 50 | 2,885 | NA |
| **Saunders et al., 2001 (USA) [46]** | 1999 - 1999 | 25,707 | 292 | 9.2 | 283 | 12,845 |
| **Shah et al., 2003 (Pakistan) ^*^[47]** | 2002 - 2002 | 386 | 3,886 | 535 | 3,351 | NA |
| **Sretrirutchai et al., 2002 (Thailand) [48]** | 1998 - 1998 | 4,751 | 568 | 228 | 340 | NA |
| **Tulsky 1998 (USA) [49]** | 1994 - 1994 | 1,659 | 59 | 13 | 46.7 | 26,851 |
| **Vieira et al., 2010 (Brazil) [50]** | 2006 - 2006 | 397 | 1,763 | 55 | 1,708 | NA |
| **White et al., 2001 (USA) [51]** | 1994 &1998 | 9,331 | 72 | 9.2 | 62.9 | 11,500 |
| **Yates et al., 2009 (UK) [52]** | NR | 5,616 | 534 | 12 | 522 | NA |
|  | | | | | | |
| ***Note: ^(a)^ As reported by WHO. NA, not applicable; NR, not reported; # Female inmates only; * Juvenile inmates only.*** | | | | | | |

**References**

1. Abebe DS, Bjune G, Ameni G, Biffa D, Abebe F (2011) Prevalence of pulmonary tuberculosis and associated risk factors in Eastern Ethiopian prisons. International Journal of Tuberculosis and Lung Disease 15: 668-673.

2. Abrahao RM, Nogueira PA, Malucelli MI (2006) Tuberculosis in county jail prisoners in the western sector of the city of Sao Paulo, Brazil. Int J Tuberc Lung Dis 10: 203-208.

3. Adib SM, Al-Takash H, Al-Hajj C (1999) Tuberculosis in Lebanese jails: prevalence and risk factors. Eur J Epidemiol 15: 253-260.

4. Aerts A, Habouzit M, Mschiladze L, Malakmadze N, Sadradze N, et al. (2000) Pulmonary tuberculosis in prisons of the ex-USSR state Georgia: results of a nation-wide prevalence survey among sentenced inmates. International Journal of Tuberculosis and Lung Disease 4: 1104-1110.

5. Assefzadeh M, Barghi RG, Shahidi Sh S (2009) Tuberculosis case--finding and treatment in the central prison of Qazvin province, Islamic Republic of Iran. East Mediterr Health J 15: 258-263.

6. Banda HT, Gausi F, Harries AD, Salaniponi FM (2009) Prevalence of smear-positive pulmonary tuberculosis among prisoners in Malawi: a national survey. Int J Tuberc Lung Dis 13: 1557-1559.

7. Banu S, Hossain A, Uddin MK, Uddin MR, Ahmed T, et al. (2010) Pulmonary tuberculosis and drug resistance in Dhaka central jail, the largest prison in Bangladesh. PLoS One 5: e10759.

8. Borja MP, Mortera LL, Palasi WA, Benedicto JP, Cadena EV, et al. (2010) Prevalence of pulmonary tuberculosis among prisoners and jail officers in the Philippines. 41st Union World Conference on Lung Health. Berlin, Germany.

9. Butler T, Levy M (1999) Mantoux positivity among prison inmates - New South Wales, 1996. Austr N ZJ Public Health 23: 185-188.

10. Carbonara S, Babudieri S, Longo B, Starnini G, Monarca R, et al. (2005) Correlates of Mycobacterium tuberculosis infection in a prison population. The European respiratory journal : official journal of the European Society for Clinical Respiratory Physiology 25: 1070-1076.

11. CDC (2003) Rapid assessment of tuberculosis in a large prison system--Botswana, 2002. MMWR Morb Mortal Wkly Rep 52: 250-252.

12. Chiang CY, Hsu CJ, Hsu PK, Suo J, Lin TP (2002) Pulmonary tuberculosis in the Taiwanese prison population. Journal of the Formosan Medical Association 101: 537-541.

13. Chigbu LN, Iroegbu CU (2010) Incidence and spread of Mycobacterium tuberculosis-associated infection among Aba Federal prison inmates in Nigeria. J Health Popul Nutr 28: 327-332.

14. Ferreira MM, Ferrazoli L, Palaci M, Salles PS, Medeiros LA, et al. (1996) Tuberculosis and HIV infection among female inmates in Sao Paulo, Brazil: a prospective cohort study. Journal of Acquired Immune Deficiency Syndromes and Human Retrovirology 13: 177-183.

15. GGD (2010) Jaarverslag 2009 Tuberculoseonderzoek in penitentiaire inrichtingen. GGD Nederland.

16. Habeenzu C, Mitarai S, Lubasi D, Mudenda V, Kantenga T, et al. (2007) Tuberculosis and multidrug resistance in Zambian prisons, 2000-2001. Int J Tuberc Lung Dis 11: 1216-1220.

17. Hussain H, Akhtar S, Nanan D (2003) Prevalence of and risk factors associated with Mycobacterium tuberculosis infection in prisoners, North West Frontier Province, Pakistan. Int J Epidemiol 32: 794-799.

18. Jittimanee SX, Ngamtrairai N, White MC, Jittimanee S (2007) A prevalence survey for smear-positive tuberculosis in Thai prisons. Int J Tuberc Lung Dis 11: 556-561.

19. Johnsen C (1993) Tuberculosis contact investigation: Two years of experience in New York City correctional facilities. American Journal of Infection Control 21: 1-4.

20. Jones TF, Craig AS, Valway SE, Woodley CL, Schaffner W (1999) Transmission of tuberculosis in a jail. Annals of Internal Medicine 131: 557-563.

21. Karabela S, Papaventsis D, Georgoulas S, Nikolaou S, Ioannidis P, et al. (2010) Epidemiological monitoring of pulmonary tuberculosis in a correctional facility population, Athens, Greece, 2005-2009. Clinical Microbiology and Infection Conference: 20th ECCMID. Vienna Austria: Blackwell Publishing Ltd.

22. Kazi AM, Shah SA, Jenkins CA, Shepherd BE, Vermund SH (2010) Risk factors and prevalence of tuberculosis, human immunodeficiency virus, syphilis, hepatitis B virus, and hepatitis C virus among prisoners in Pakistan. Int J Infect Dis 14 Suppl 3: e60-66.

23. Kiter G, Arpaz S, Keskin S, Sezgin N, Budin D, et al. (2003) Tuberculosis in Nazilli District Prison, Turkey, 1997-2001. International Journal of Tuberculosis and Lung Disease 7: 153-158.

24. Koo DT, Baron RC, Rutherford GW (1997) Transmission of Mycobacterium tuberculosis in a California State Prison, 1991. Am J Public Health 87: 279-282.

25. Leung CC, Chan CK, Tam CM, Yew WW, Kam KM, et al. (2005) Chest radiograph screening for tuberculosis in a Hong Kong prison. International Journal of Tuberculosis and Lung Disease 9: 627-632.

26. Levy MH, Butler TG, Zhou J (2007) Prevalence of Mantoux positivity and annual risk of infection for tuberculosis in New South Wales prisoners, 1996 and 2001. N S W Public Health Bull 18: 119-124.

27. MacIntyre CR, Carnie J, Randall M (1999) Risk of transmission of tuberculosis among inmates of an Australian prison. Epidemiol Infect 123: 445-450.

28. MacIntyre CR, Kendig N, Kummer L, Birago S, Graham NM (1997) Impact of tuberculosis control measures and crowding on the incidence of tuberculous infection in Maryland prisons. Clin Infect Dis 24: 1060-1067.

29. Martin Sanchez V, Gonzalez P, Cayla JA, Mirabent J, Canellas J, et al. (1994) Case-finding of pulmonary tuberculosis on admission to a penitentiary centre. Tubercle and Lung Disease 75: 49-53.

30. Martin Sanchez VM, Alvarez-Guisasola F, Cayla JA, Alvarez JL (1995) Predictive factors of Mycobacterium tuberculosis infection and pulmonary tuberculosis in prisoners. International Journal of Epidemiology 24: 630-636.

31. Martin Sanchez VM, Guerra JM, Cayla JA, Rodriguez JC, Blanco MD, et al. (2001) Incidence of tuberculosis and the importance of treatment of latent tuberculosis infection in a Spanish prison population. Int J Tuberc Lung Dis 5: 926-932.

32. McLaughlin SI, Spradling P, Drociuk D, Ridzon R, Pozsik CJ, et al. (2003) Extensive transmission of Mycobacterium tuberculosis among congregated, HIV-infected prison inmates in South Carolina, United States. Int J Tuberc Lung Dis 7: 665-672.

33. Mor Z, Adler A, Leventhal A, Volovic I, Rosenfeld E, et al. (2008) Tuberculosis behind bars in Israel: policy making within a dynamic situation. Isr Med Assoc J 10: 202-206.

34. Nduaguba IP, Brannan G, Shubrook J (2010) Evaluation of identifying tuberculosis infection and disease in a rural institutionalized population. Osteopathic Family Physician 2: 10-13.

35. Noeske J, Kuaban C, Amougou G, Piubello A, Pouillot R (2006) Pulmonary tuberculosis in the Central Prison of Douala, Cameroon. East Afr Med J 83: 25-30.

36. Noeske J, Ndi N, Mbondi S (2011) Controlling tuberculosis in prisons against confinement conditions: a lost case? Experience from Cameroon. Int J Tuberc Lung Dis 15: 223-227, i.

37. Nyangulu DS, Harries AD, Kang'ombe C, Yadidi AE, Chokani K, et al. (1997) Tuberculosis in a prison population in Malawi. Lancet 350: 1284-1287.

38. Payanandana V, Donram W, Jittimanee S, Piatanya P, Ngamtriri N, et al. (2002) Tuberculosis among prison inmate population, Thailand: a survey of tuberculosis burden, factors associated with development of disease, and effectiveness of supervised intermittent regimens. Thai J Tuberc Chest Dis 23: 167-177.

39. Puisis M, Feinglass J, Lidow E, Mansour M (1996) Radiographic screening for tuberculosis in a large urban county jail. Public Health Reports 111: 330-334.

40. Rao NA (2004) Prevalence of pulmonary tuberculosis in Karachi central prison. Journal of the Pakistan Medical Association 54: 413-415.

41. Rutz HJ, Bur S, Lobato MN, Baucom S, Bohle E, et al. (2008) Tuberculosis control in a large urban jail: discordance between policy and reality. J Public Health Manag Pract 14: 442-447.

42. Sanchez A, Diuana V, Romano E, Pires J, Espinola AB, et al. (2010) Tuberculosis control in prisons: Impact of intensive education and screening at entry. 41st Union World Conference on Lung Health. Berlin, Germany.

43. Sanchez A, Gerhardt G, Natal S, Capone D, Espinola A, et al. (2005) Prevalence of pulmonary tuberculosis and comparative evaluation of screening strategies in a Brazilian prison. International Journal of Tuberculosis and Lung Disease 9: 633-639.

44. Sanchez A, Larouze B, Espinola AB, Pires J, Capone D, et al. (2009) Screening for tuberculosis on admission to highly endemic prisons? The case of Rio de Janeiro State prisons. Int J Tuberc Lung Dis 13: 1247-1252.

45. Sanchez A, Massari V, Gerhardt G, Cesconi V, Espinola AB, et al. (2010(a)) Tuberculosis control in highly endemic prisons: impact of X-ray at entry and mass screening. 41st Union World Conference on Lung Health. Berlin, Germany.

46. Saunders DL, Olive DM, Wallace SB, Lacy D, Leyba R, et al. (2001) Tuberculosis screening in the federal prison system: an opportunity to treat and prevent tuberculosis in foreign-born populations. Public Health Reports 116: 210-218.

47. Shah SA, Mujeeb SA, Mirza A, Nabi KG, Siddiqui Q (2003) Prevalence of pulmonary tuberculosis in Karachi juvenile jail, Pakistan. Eastern Mediterranean Health Journal 9: 667-674.

48. Sretrirutchai S, Silapapojakul K, Palittapongarnpim P, Phongdara A, Vuddhakul V (2002) Tuberculosis in Thai prisons: magnitude, transmission and drug susceptibility. International Journal of Tuberculosis and Lung Disease 6: 208-214.

49. Tulsky JP, White MC, Dawson C, Hoynes TM, Goldenson J, et al. (1998) Screening for tuberculosis in jail and clinic follow-up after release. Am J Public Health 88: 223-226.

50. Vieira AA, Ribeiro SA, de Siqueira AM, Galesi VM, dos Santos LA, et al. (2010) Prevalence of patients with respiratory symptoms through active case finding and diagnosis of pulmonary tuberculosis among prisoners and related predictors in a jail in the city of Carapicuiba, Brazil. Rev Bras Epidemiol 13: 641-650.

51. White MC, Tulsky JP, Portillo CJ, Menendez E, Cruz E, et al. (2001) Tuberculosis prevalence in an urban jail: 1994 and 1998. Int J Tuberc Lung Dis 5: 400-404.

52. Yates S, Story A, Hayward AC (2009) Screening prisoners for tuberculosis: What should the UK do? Thorax Conference: British Thoracic Society, BTS Winter Meeting 2009. London United Kingdom.: BMJ Publishing Group.
